# Leveraging ligand-based proton and electron transfer for aerobic reactivity and catalysis

**DOI:** 10.1039/d4sc03896g

**Published:** 2024-09-09

**Authors:** Kate A. Jesse, John S. Anderson

**Affiliations:** a Los Alamos National Laboratory Los Alamos NM 87545 USA kjesse@lanl.gov; b Department of Chemistry, The University of Chicago Chicago Illinois 60637 USA jsanderson@uchicago.edu

## Abstract

While O_2_ is an abundant, benign, and thermodynamically potent oxidant, it is also kinetically inert. This frequently limits its use in synthetic transformations. Correspondingly, direct aerobic reactivity with O_2_ often requires comparatively harsh or forcing conditions to overcome this kinetic barrier. Forcing conditions limit product selectivity and can lead to over oxidation. Alternatively, O_2_ can be activated by a catalyst to facilitate oxidative reactivity, and there are a variety of sophisticated examples where transition metal catalysts facilitate aerobic reactivity. Many efforts have focused on using metal–ligand cooperativity to facilitate the movement of protons and electrons for O_2_ activation. This approach is inspired by enzyme active sites, which frequently use the secondary sphere to facilitate both the activation of O_2_ and the oxidation of substrates. However, there has only recently been a focus on harnessing metal–ligand cooperativity for aerobic reactivity and, especially, catalysis. This perspective will discuss recent efforts to channel metal–ligand cooperativity for the activation of O_2_, the generation and stabilization of reactive metal–oxygen intermediates, and oxidative reactivity and catalysis. While significant progress has been made in this area, there are still challenges to overcome and opportunities for the development of efficient catalysts which leverage this biomimetic strategy.

## Abstract 2

O_2_ is an attractive oxidant but it is also kinetically inert which limits its use in synthetic transformations. Metal–ligand cooperativity is a promising biomimetic strategy to activate O_2_ for oxidative reactivity and catalysis.

## Graphical abstract

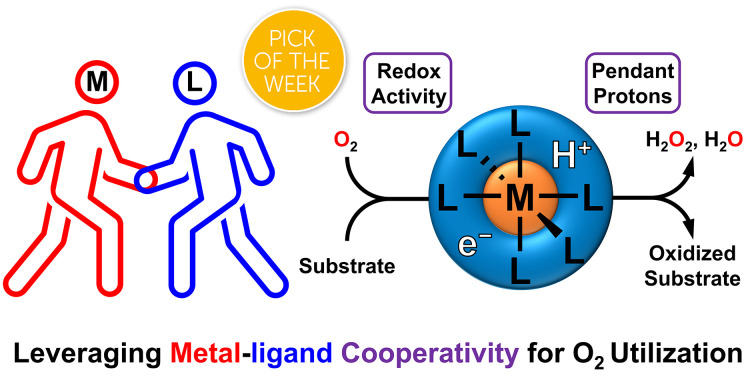

## 1 Introduction

O_2_ is an abundant, inexpensive, and thermodynamically powerful oxidant. This thermodynamic potency is perhaps most simply illustrated by the formal electrochemical potential of 1.23 V *vs.* SHE for O_2_ reduction.^1^ Importantly, this net reduction of O_2_ results in water as a benign byproduct. Despite these advantages, O_2_ is comparatively kinetically inert due to its triplet ground spin state. This triplet ground state leads to inhibited reactivity with many compounds, such as most organic substrates that typically have singlet spin states.^2^ This kinetic inertness is also illustrated by the bond dissociation free energy (BDFE) of ∼51 kcal mol^−1^ in solvent for the first proton/electron in the reduction of O_2_ to water.^3^ To overcome these spin-forbidden processes, and to participate in oxidative reactivity, O_2_ must enter the higher energy singlet spin state.^2^ This means that direct aerobic reactivity often requires forcing conditions, such as the high temperatures used for combustion, or activation *via* a catalyst. Transition metal complexes feature prominently in aerobic catalysis, which has led to a wide array of elegant examples in which O_2_ is activated for catalytic transformations. The Wacker oxidation, which was developed in 1956, is one classic example that uses co-catalytic Pd and Cu to perform the oxidation of ethylene to acetaldehyde with O_2_ as a terminal oxidant.^4–6^ Cu is essential for this process as it helps with mediating efficient redox exchange between Pd and O_2_. Alternatively, a supporting ligand can be similarly used to facilitate proton/electron transfer and subsequent O_2_ activation. For example, Stahl and coworkers have investigated the utility of organic cocatalysts such as aminoxyl or quinone compounds that work in conjunction with metal salts to facilitate aerobic oxidation chemistry.^7–16^ There has also been extensive research into metal-free aerobic reactivity, primarily using aminoxyl compounds.^17–25^

While there have been many successes in this area, it is noteworthy that Nature activates O_2_ through elaborate active sites that coordinate transition metal centers with a carefully positioned organic secondary coordination sphere. Secondary coordination spheres in active sites for aerobic catalysis nearly always feature themes such as hydrogen bonding motifs, redox-active cofactors for electron transfer, and proton shuttling machinery, all of which aid in O_2_ activation.^26–33^ In many cases this activation can lead to more thermodynamically potent oxidants, such as terminal oxo complexes, through the controlled addition of H-atom equivalents. Thus, the elaborate “ligand” of enzymatic secondary coordination spheres is critical for the activation of O_2_ and subsequently efficient and selective aerobic oxidations.

Despite the biological ubiquity of coupling transition metals with tailored secondary coordination spheres to facilitate aerobic reactivity, there has only recently been an allied focus on leveraging these same influences in synthetic complexes.^13,34^ Major advances include hydrogen bonding ligands,^35–38^ proton shuttling ligands,^39–46^ and redox-active ligands for cooperative electron transfer.^47–60^ While these approaches are now broadly used in the design of new synthetic catalysts, their application for O_2_ activation, particularly aerobic catalysis, is still nascent. Supporting ligand scaffolds that can cooperatively transfer both protons and electrons to activate O_2_ for aerobic reactivity in a biomimetic manner are even more rare.

**Fig. 1:**
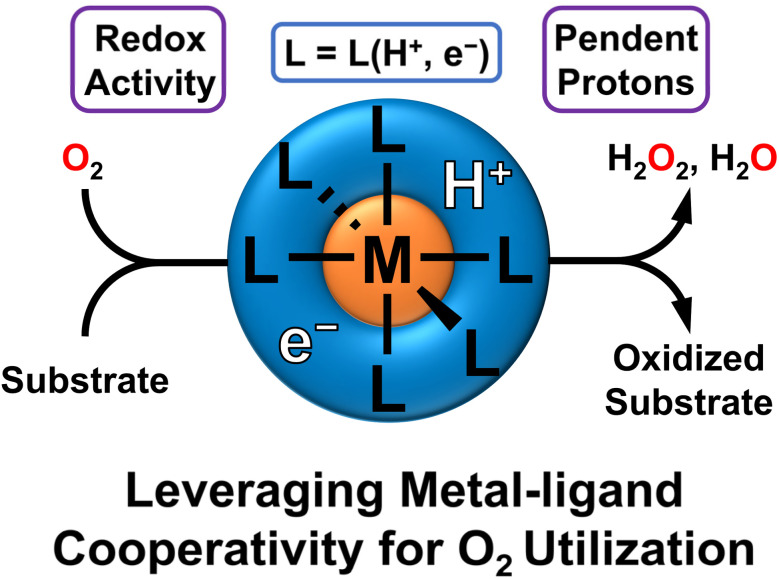
Metal–ligand cooperativity that incorporates redox-active ligands and pendent H-equivalents in the secondary sphere can activate O_2_ for oxidative reactivity with substrates. This enables the ligand to serve as a proton and electron source.

This perspective will cover recent advances in leveraging metal–ligand cooperativity for O_2_ activation and aerobic reactivity (Fig. 1). We will particularly focus on systems that can shuttle both protons and electrons to O_2_, beginning with examples where this cooperativity enables the characterization of well-defined intermediates which feature O_2_ binding or reduction. We will then focus on examples which display stoichiometric or catalytic oxidations. Last, we will summarize prospects and challenges, laying out key directions for development and investigation as this field develops. One clear conclusion is that this area holds promise for developing sustainable oxidation reactions, with exciting developments likely coming in the near future.

## 2 Metal–ligand cooperativity enabling the activation or reduction of O_2_

**Fig. 2:**
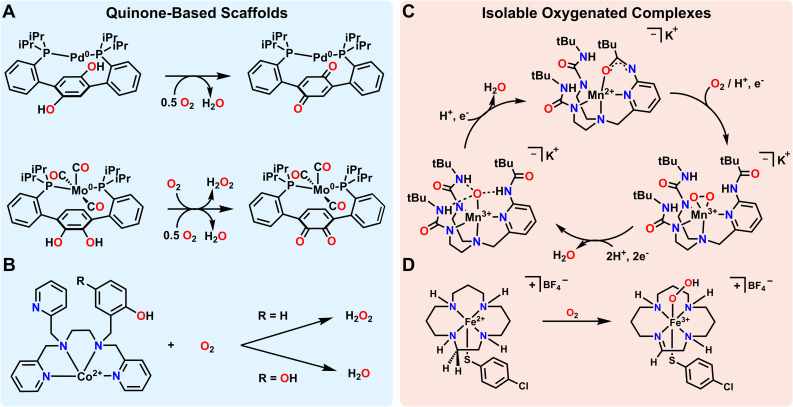
Metal–ligand cooperative transfer of protons and electrons enabling the activation of O_2_. (A and B) Quinone-based non-innocent ligands that can donate protons and electrons to reduce O_2_. (C) A non-innocent ligand stabilizes metal–oxygen intermediates and acts as a proton shuttle to reduce O_2_ to water in the presence of external reductant. (D) Protons and electrons from a non-innocent ligand produce a spectroscopically characterizable Fe–hydroperoxo complex.

Metal–ligand cooperativity involving ligand-stored protons and electrons has been shown to facilitate the formation and subsequent reduction of various metal–oxygen species such as metal-oxos and metal hydroperoxos from O_2_. Again, this is a common mode of reactivity and O_2_ activation in biology, but it has also become a useful approach in synthetic systems. Quinoidal motifs are some of the classic building blocks used in redox non-innocent ligands, and ligands with incorporated quinones/quinols can further provide an accessible proton and electron source for O_2_ reduction. Agapie and coworkers effectively used such a quinone based ligand scaffold with Pd to facilitate O_2_ reduction to water (Fig. 2A). The diphosphine hydroquinone ligand is able to donate 2 electrons and 2 protons towards O_2_ reduction. When a Pd atom is coordinated to the ligand, O_2_ reduction is found to proceed *via* a side-on η^2^-peroxo complex, the formation of which can be observed by low temperature ultraviolet-visible (UV-vis) spectroscopy. Low temperature ^31^P nuclear magnetic resonance (NMR) spectroscopy of this species supports the formation of an asymmetric complex, and low temperature infra-red (IR) spectroscopy shows no indication of a C=O double bond stretch, indicating that the side on Pd η^2^-peroxo complex forms prior to the involvement of the backbone quinone. While this first intermediate observed by UV-vis spectroscopy is long-lived at low temperature, the second intermediate generated upon warming decays more rapidly. Spectroscopic data of this second species suggests a Pd^2+^ complex coordinated to a semiquinone ligand, but concrete assignment of the partially hydrogenated oxygen ligand was not obtained due to the transient nature of this intermediate. The reduction of O_2_ to H_2_O as a final product was confirmed by ^1^H NMR spectroscopic analysis of the reaction volatiles after transferring to a J. Young tube.^61^

Agapie and coworkers have also illustrated the utility of a related isomeric quinone-based ligand scaffold on Mo complexes for a similar reduction of O_2_ to water (Fig. 2A). In this case it was found that activation of O_2_ led to initial reduction to H_2_O_2_ rather than H_2_O. H_2_O_2_ then putatively interacts with a second equivalent of the Mo complex for the net 4e^−^ reduction to H_2_O to occur. This illustrates how both the ligand and the metal can influence the selectivity of reductions, even in closely related systems. As a final note, while this perspective focuses on the reduction of O_2_ with protons and electrons, Mo complexes supported by this general ligand framework, but with Si- or B-functionalized catecholate motifs, make (R_2_SiO)_*n*_ and (R_2_BO)_*n*_ byproducts rather than H_2_O as the final reaction product.^62^ Regardless of the terminal O-accepting reagent, all of these examples illustrate how redox-active quinone-based ligands can facilitate selective reduction of O_2_ to H_2_O.

While the above examples rely on the incorporation of quinone/quinol moieties directly into the ligand backbone, the use of a pendent quinol on a ligand arm has also been shown to facilitate the reduction and/or activation of O_2_. In particular, Goldsmith and coworkers have shown that appending such a quinol group to a chelating amine/pyridine scaffold facilitates superoxide dismutase (SOD) like reactivity with either Mn^2+^ or Zn^2+^.^63,64^ Mn^2+^ complexes with a ligand featuring one pendent phenol mediate SOD reactivity *via* an outer-sphere mechanism. However, when the ligand features one or two pendent quinols, the reaction accelerates *via* an inner-sphere mechanism.^63^ This quinol assisted inner-sphere pathway can also be leveraged to render a redox-inactive metal ion, Zn^2+^, active for SOD catalysis.^64^ Beyond this example, Goldsmith and coworkers have also investigated the electrochemical reduction of O_2_ using a Co^2+^ metal center bound to the same ligand framework (Fig. 2B). They found that when a pendent quinol rather than a pendent phenol was incorporated into the ligand scaffold, the product selectivity and mechanism shifts from a 2 electron, 2 proton reduction of O_2_ to form H_2_O_2_ to a 4 electron, 2 proton reduction of O_2_ to form H_2_O.^65^ This switch demonstrates that inclusion of groups that donate both electrons and protons not only enables catalytic reactivity, but also provides determinative control over selectivity in some processes. This conclusion meshes with other observations in biological and synthetic systems. As one example, Machan and coworkers and Stahl and coworkers have both shown the utility of 1,4-hydroquinone/benzoquinone as a cocatalyst in facilitating the reduction of O_2_ to H_2_O electrocatalytically.^9,66^ While the 1,4-hydroquinone/benzoquinone group is not directly incorporated into the ligand backbone in these cases, the electrocatalytic reaction mechanism requires the close association of 1,4-hydroquinone/benzoquinone to produce H_2_O as the final product. As with the Co example above, omission of 1,4-hydroquinone as a cocatalyst in this example from Stahl and coworkers^9^ instead results in the preferential reduction of O_2_ to H_2_O_2_.

An alternate strategy for metal–ligand cooperativity is the use of the ligand to create a secondary sphere that stabilizes reactive oxygenated intermediates through hydrogen bonding interactions. The use of H-bonding or proton shuttling ligands has been of great recent interest.^67^ While these scaffolds do not strictly shuttle electrons, there are several ligand scaffolds that feature conjugated arms that may potentially also be redox-active. As such, some select cases are also highlighted here. As one example among many,^35–38,68,69^ Borovik and coworkers demonstrated how designing a secondary sphere to stabilize intermediates and shuttle proton equivalents enables the catalytic reduction of O_2_. They were able to synthesize a side on Mn-peroxo species and Mn-oxo species stabilized by hydrogen bonding interactions (Fig. 2C). Characterization of these intermediates proved crucial to the development of a catalytic cycle converting O_2_ to H_2_O at room temperature.^70^ Catalysis was only observed with the addition of hydrazine or diphenylhydrazine as a terminal reductant. Fout and coworkers utilized a similar strategy to stabilize Fe^3+^-oxo or Fe^3+^-hydroxo complexes formed *via* reactivity between an Fe complex and O_2_.^35^ Both complexes are stabilized by hydrogen bonding interactions from the ligand, and the ligand shuttles protons to the O_2_ substrate.^67^ Szymczak and coworkers have also used hydrogen bonding motifs to stabilize a Zn-peroxo dimer formed from reactivity with O_2_ in the presence of cobaltocene. However, we note that neither protons nor electrons are shuttled by the ligand for O_2_ reduction in this case.^69^ The fact that no H-equivalents are transferred to O_2_ makes the formation of the Zn-peroxo dimer reversible with added oxidant, such as ceric ammonium nitrate or iodobenzene dichloride. Another related example comes from Garcia–Bosch and coworkers. They recently reported a Cu complex that uses hydrogen bonding interactions from the ligand to stabilize and reduce O_2_ to an axial hydroperoxo ligand. However, in this case, the terminal H-atom on the hydroperoxo ligand is abstracted from the ligand of a second Cu complex rather than the oxidized Cu complex itself.^71^ In all examples, hydrogen bonding interactions help to stabilize reactive intermediates, with some examples also invoking H-atom abstraction and proton shuttling for catalytic reactivity. While definitive evidence for ligand-based redox reactivity is less obvious in most of these cases, it is possible, if not likely, that the conjugated arms in these or related scaffolds might also be non-innocent under the right conditions or with appropriate synthetic modifications.

It is noteworthy that while the quinone/quinol systems discussed above formally transfer H-atoms, the H-bonding examples feature N-based ligands which do not as explicitly exhibit net H-atom reactivity. However, there are other ligands with N–H's that more clearly shuttle reducing equivalents to O_2_. David Goldberg and coworkers found that they could use a macrocyclic tetraamine ligand combined with electronic effects from a trans thiolate ligand to stabilize an Fe^3+^-hydroperoxo complex (Fig. 2D).^72^ Here, the electron and proton equivalents came from the ligand scaffold *via* desaturation to form an imine. This complex can be generated at −78 °C, and was characterized by UV-visible spectroscopy, electron paramagnetic resonance (EPR) spectroscopy, and electrospray ionization mass spectrometry (EI-MS). Using thiolate ligands for stabilizing Fe–oxygen intermediates has been a topic of some interest due to the presence of this motif in enzyme active sites such as superoxide reductase (SOR) or cytochrome P450.^73,74^ However, these biomimetic H_*n*_O_*y*_ complexes are rarely generated directly from O_2_ since their formation requires the movement of both protons and electrons. Instead, Fe-hydroperoxo complexes are often generated from exogenous reducing and acid equivalents.^75–84^ The reactivity observed in this case would be impossible without metal–ligand cooperativity arising from desaturation of the macrocyclic ligand that facilitates the transfer of both electrons and protons.

**Fig. 3:**
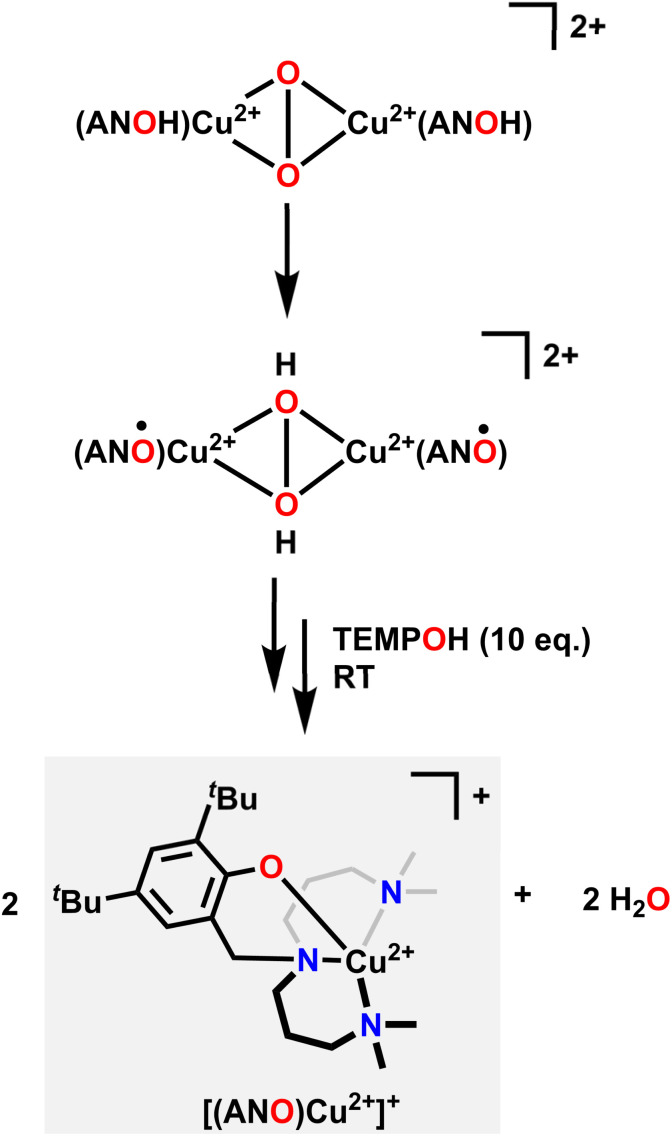
Mechanism of O_2_ capture, hydrogenation, then release of water upon reacting with TEMPOH.

Similar in structure to quinols, pendent phenol functionalities on the ligand backbone can act as a proton and electron source for the hydrogenation of O_2_. Karlin and coworkers used a Cu system with an aliphatic amine pincer ligand and a pendent phenol to capture O_2_ as a bis(μ-oxo)Cu_2_^2+^ dimer at −135 °C.^85^ The pendent phenol group on each ligand then donates one proton and one electron to each O atom, resulting in a bis(μ-hydroxo)Cu_2_^2+^ dimer where each Cu center features a ligand-based radical on the O atom of the pendent phenol. These ligand-based radicals can be detected with EPR spectroscopy by a characteristic signal at *g* = 2.00. Upon warming in the presence of excess 1-hydroxy-2,2,6,6-tetramethylpiperidine (TEMPOH) as an H-atom source, this dimer releases two equivalents of H_2_O and forms a Cu^2+^ complex with an overall +1 charge, where the Cu center is now coordinated to the NNN amine pincer ligand as well as the O in the pendent phenol (Fig. 3). This reactivity mimics the reactivity observed in the active site of oxy-tyrosinase, which catalyses the regioselective hydroxylation of monophenols with a *para* substitution to catechols.^86,87^

In each of the examples presented in this section, metal–ligand cooperativity facilitates the shuttling of proton and electron equivalents to O_2_ resulting in its reduction. Despite this general reactivity paradigm, the products of these reactions are variable depending on the system, with H_2_O, H_2_O_2_, or stabilized metal–oxygen complexes (*i.e.* hydroperoxo species) that can be spectroscopically characterized all being commonly formed. While these examples demonstrate the utility of incorporating redox active ligands with pendent protons for the hydrogenation of O_2_, there is still a lack of clarity on what exact design features facilitate the formation of different products. Simple topics of future investigation might reasonably include examining how the positioning of H-donors dictates product selectivity, or how the thermodynamic properties of these donors (BDFE, acidity, *etc.*) can be tuned for desired reactivity. Beyond these fundamental studies, there is also great potential in harnessing the thermodynamic potential of O_2_ for subsequent substrate oxidations after activation. Indeed, the next sections of this perspective focus on recent advances in these areas.

## 3 Metal–ligand cooperativity enabling the activation of O_2_ for alcohol oxidation

Alcohol oxidation is a widely used transformation in synthetic chemistry. Metal–ligand cooperativity can facilitate alcohol oxidation reactions by either forming an oxygenated intermediate that serves as the active oxidant in the key bond forming/breaking steps or as an inexpensive and benign terminal oxidant for the regeneration of an oxidized transition metal intermediate that instead directly oxidizes the substrate, as seen in Wacker chemistry.^4,5^ In both instances, O_2_ acts as a formal H-atom acceptor, but there are key mechanistic differences whether an *in situ* oxygenated intermediate acts as the oxidant or whether O_2_ simply acts as a regenerating terminal oxidant.

**Fig. 4:**
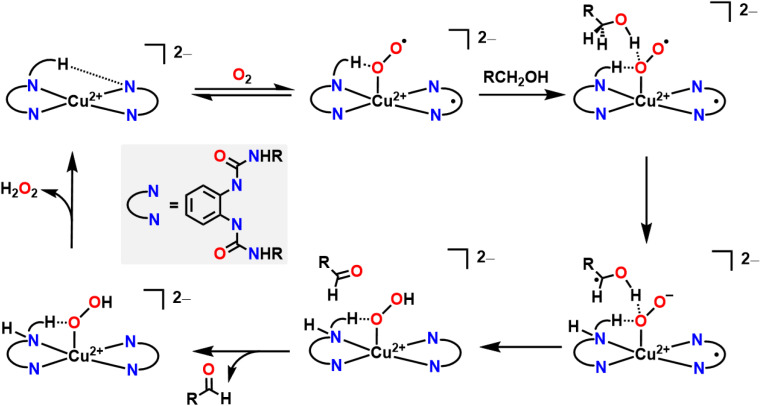
Mechanism of alcohol oxidation where O_2_ participates in the mechanism in conjunction with ligand non-innocence. O_2_ generates the Cu-superoxo that enables alcohol oxidation before being reduced *via* metal–ligand cooperativity.

**Fig. 5:**
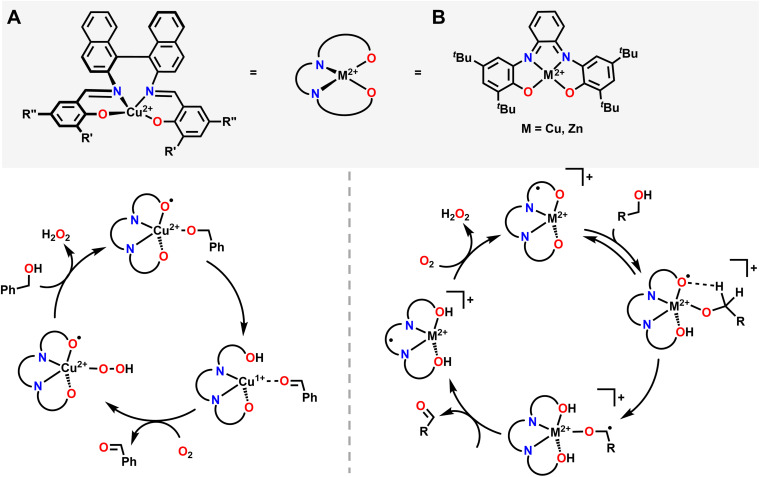
(A) Proposed mechanism of alcohol oxidation reported by Stack and coworkers closely mimicking the ping-pong mechanism observed in GAO. O_2_ participates in the mechanism in conjunction with ligand non-innocence. O_2_ generates a Cu-hydroperoxo and displaces the oxidized substrate, before being reduced *via* metal–ligand cooperativity to H_2_O_2_. (B) Proposed mechanism of primary alcohol oxidation reported by Wieghardt and coworkers. In this case O_2_ serves as a terminal oxidant, allowing the starting metal catalyst to regenerate.

A good example where O_2_ generates an active oxygenated transition metal intermediate that performs alcohol oxidation comes from Garcia–Bosch and coworkers (Fig. 4).^88^ They use a ureanyl functionalized 1,2-phenylenediamido Cu^2+^ platform where the diureanyl ligand can formally store both protons and electrons as well as provide H-bonding interactions. This platform enables the stabilization of a superoxo intermediate *via* H-bonding interactions from the ligand. This transiently stabilized Cu-superoxo then serves as the active oxidant for the oxidation of primary alcohols to aldehydes. The ligand further facilitates the reaction by mediating the movement of proton and electron equivalents during the dehydrogenation of the alcohol and subsequent H-transfer to the coordinated O_2_ molecule resulting in both aldehyde and H_2_O_2_ as the terminal reaction products. Throughout the course of this reaction mechanism, Cu remains in a 2+ state. This means that the Cu^2+^ center does not participate in the alcohol oxidation reaction as an electron source, but rather as a docking site for the primary alcohol/O_2_ to coordinate such that the ligand secondary sphere can mediate the resulting oxidation and reduction respectively. Interestingly, this suggests that transition metal centers, which are frequently considered as redox mediators in many catalytic cycles, can instead be tuned/utilized as simple Lewis acid sites for coordination if a suitably redox-active supporting ligand is used.^64,89–91^ This is similar to how classic reactions in organic chemistry, such as the Sharpless epoxidation, proceed through use of a catalyst that acts as a Lewis acid.^92^ Additionally, the reduction of O_2_ to H_2_O_2_ allows for the regeneration of the starting complex and thus allows for a catalytic process. This work is in contrast to the ping-pong mechanism seen in galactose oxidase (GAO), where a primary alcohol is first oxidized to an aldehyde, then O_2_ is reduced to H_2_O_2_ as the terminal regenerating oxidant to reform the active catalyst. The amino acids within the GAO active site act as proton acceptors, whereas Cu plays a more active role in the catalytic cycle of GAO, by acting as an electron acceptor/donor. This ping-pong mechanism has been more closely reproduced by Stack and coworkers (Fig. 5A).^93^ Here the authors were able to synthesize a Cu complex and related intermediates in the catalytic oxidation of primary alcohols and demonstrate pathways that match those proposed for GAO. In their Cu system, alcohol oxidation occurs first with the ligand scaffold acting as an H-atom acceptor, O_2_ reduction to H_2_O_2_ occurs second, and the Cu^2+^ center is reversibly reduced to Cu^1+^ during the course of the catalytic cycle.^93^ As such, this example from Stack serves as a more faithful biomimetic Cu system for the mechanism observed in GAO where O_2_ serves as a regenerating oxidant.

Pioneering work from Wieghardt and coworkers demonstrated that different mononuclear Cu and Zn complexes featuring an ONNO ligand structurally similar the ligand used by Stack and coworkers were also able to facilitate alcohol oxidation of primary and benzylic alcohols using O_2_ as an oxidant to regenerate the starting metal complex catalytically and release H_2_O_2_ (Fig. 5B).^94,95^ Mechanistic and kinetic studies of their system showed evidence for H-atom abstraction by the ligand from the coordinated alcohol, providing evidence for ligand noninnocence and metal–ligand cooperativity during the alcohol oxidation reaction.

Another early example of a Cu complex facilitating alcohol oxidation in conjunction with O_2_ reduction comes from Urch, Brown, and coworkers.^96,97^ In their system, CuCl was mixed with phenanthroline (phen), a substituted azo compound, such as di-*tert*-butylazodicarboxylate (DBAD), an alcohol, O_2_, and K_2_CO_3_, to form a Cu^1+^ complex that facilitates alcohol oxidation *via* hydrogen transfer from the coordinated alcohol to the coordinated azo ligand. Upon release of the aldehyde or ketone, O_2_ then coordinates to form a Cu^2+^ dimer with a bridging peroxo ligand. The peroxo ligand is then homolytically cleaved when heated to putatively form a Cu^2+^-oxyl complex. This then abstracts an H-atom from both the substituted hydrazine ligand and the alcohol substrate to release H_2_O and restart the catalytic cycle. Interestingly, although this is one of the earliest examples invoking metal–ligand cooperativity for alcohol oxidation with a Cu complex where O_2_ is used as a terminal oxidant, it is also one of the examples with the widest substrate scope. Urch, Brown, and coworkers found that this system is able to catalytically oxidize primary, allylic, and benzylic alcohols.^97^

**Fig. 6:**
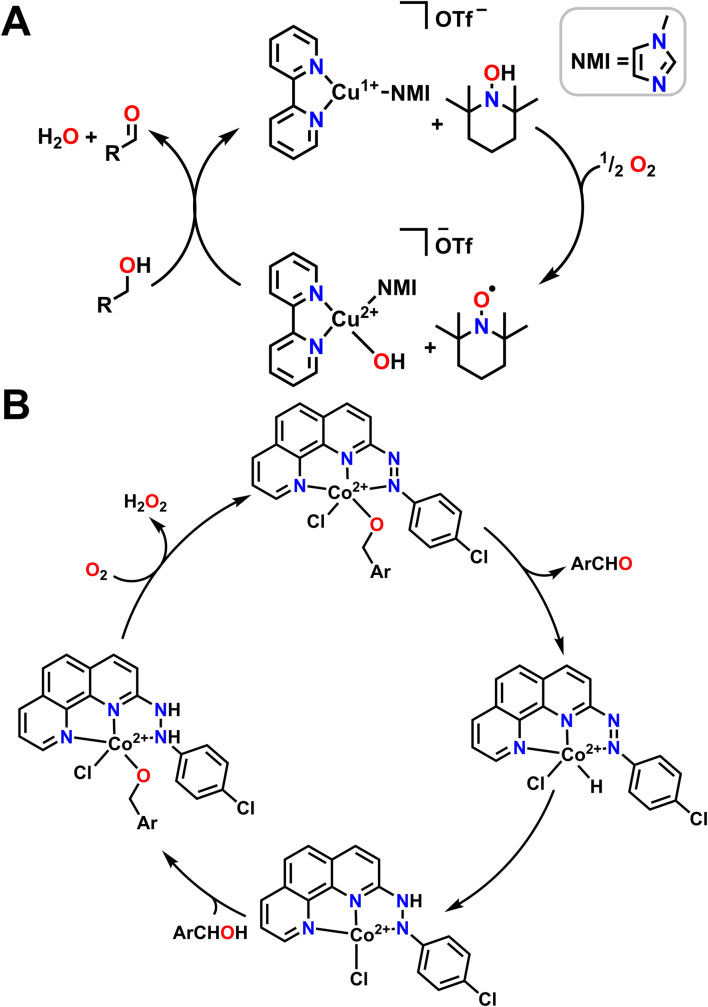
Mechanisms of alcohol oxidation where O_2_ participates in the mechanism in conjunction with ligand non-innocence. (A) O_2_ is reduced with an Cu complex and external organic compound that serves as a proton and electron source to regenerate the active molecule for alcohol oxidation. NMI coordinates through the less sterically hindered N atom. (B) O_2_ regenerates starting metal complex.

While there are entropic and orientational advantages to having a tethered ligand for H-shuttling, these scaffolds can frequently be difficult to synthesize or tune, which limits their application in organic methodology. An alternative strategy that has been employed to great effect is the use of metal complexes in conjunction with an exogenous organic cocatalyst that acts as a proton/electron reservoir. Stahl and coworkers have been particularly active in this area with extensive applications of Cu bipyridine (bpy) complexes with (2,2,6,6-tetramethylpiperidin-1-yl)oxidanyl (TEMPO) as a cocatalyst for alcohol oxidation (Fig. 6A).^98^ They find that half an equivalent of O_2_ and one equivalent of TEMPO are necessary to generate a Cu^2+^ hydroxide complex which can then bind alcohol. TEMPO then serves as an H-atom acceptor in tandem with the Cu^2+^ center serving as a one e^−^ oxidant to regenerate the starting catalyst and generate the aldehyde product. Other aminoxyl co-catalysts are also competent for this reactivity; when TEMPO is switched out for 9-azabicyclo[3.3.1]nonane N-oxyl (ABNO), the oxidation of both primary and secondary alcohols becomes accessible.^99^ The transition metal used can also be changed; Stahl and coworkers have also had success using Fe(NO_*x*_) catalysts instead of Cu(bpy) in conjunction with aminoxyl compounds.^100^

While both Cu and Fe are frequently used for oxidative chemistry with O_2_, particularly in biological or biomimetic contexts, other first row transition metals with redox active ligands and pendent protons can also use O_2_ for alcohol oxidation. Many of these examples again leverage metal–ligand cooperativity. As one example, the Paul group used 2-(4-chlorophenylazo)-1,10-phenanthroline ligand with Co^2+^ to catalyse alcohol oxidation with benzyl alcohols (Fig. 6B).^101^ The proposed reaction mechanism begins by coordinating the alcohol and releasing HCl. The catalytic cycle then proceeds *via* β-hydride elimination from the bound alcohol to release an aldehyde or a ketone while simultaneously transferring the hydride onto Co^2+^ to form a transient Co hydride intermediate. The hydride ligand can then transfer onto a diazene fragment of the supporting ligand before a new alcohol equivalent coordinates to Co^2+^ as an alkoxide, with the ligand accepting a proton. An O_2_ molecule then oxidizes the ligand, removing a formal H_2_ equivalent and releasing H_2_O_2_ to reform the starting complex in the catalytic cycle (Fig. 6B).

The examples discussed in this section offer some general mechanistic paradigms for catalytic systems featuring both metal–ligand cooperativity and O_2_. Generally, O_2_ is either used in the generation of the active oxidant, or it is used to regenerate the active catalyst. In both cases, hydrogenation of O_2_ occurs in addition to the targeted alcohol oxidation as part of the catalytic cycle. The production of H_2_O or H_2_O_2_ can result in its own challenges since these molecules may participate in side-reactions or competitive coordination, particularly as their relative concentration increases. This is a challenge that will need to be addressed in any future large scale catalytic process, particularly in examples which generate H_2_O_2_.

## 4 Metal–ligand cooperativity enabling the activation of O_2_ for O-atom transfer and other dehydrogenation reactions

**Fig. 7:**
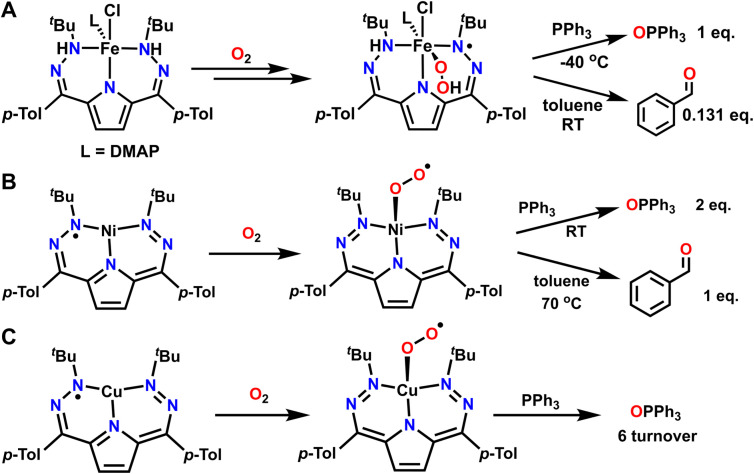
Exposure to O_2_ forms metal–oxygen complexes that can then facilitate O-atom transfer reactions to phosphines and C–H bonds *via* metal ligand cooperativity. (A) Reactivity of a fully reduced and hydrogenated Fe(DHP)–H_2_ complex. (B) Formation and reactivity of a Ni(DHP)-superoxo complex. (C) Formation and reactivity of a Cu(DHP)-superoxo complex.

Beyond using O_2_ as a reagent to drive overall oxidative reactivity, metal–ligand cooperativity can also facilitate using O_2_ as an O-atom transfer reagent. This typically occurs by first forming an oxygenated metal complex such as metal-superoxo, -hydroperoxo, or -oxo species which then acts as the active O-atom transfer reagent. Several examples where O_2_ can be used for net O-atom transfer have come from our laboratory using a dihydrazonopyrrole (DHP) based ligand scaffold and various first row transition metals. This ligand scaffold has the ability to reversibly donate or accept two protons and two electrons, while additional electron equivalents can be accessed from the metal center itself.^102,103^ This unique redox flexibility directly aids in the activation of O_2_, in many cases through spectroscopically observable intermediates. Metalation with Fe^2+^ enables the isolation and characterization of a reduced and protonated DHP complex with a formal H_2_ equivalent stored on the ligand backbone. Assignment of this complex is supported by suite of spectroscopic techniques, including single crystal X-ray diffraction (SXRD) and IR spectroscopy, both of which support the presence of ligand-based N–H protons. In the presence of O_2_, this complex reacts to form a transiently stable intermediate which can be assigned as an Fe^3+^-hydroperoxo complex with a ligand based DHP radical (Fig. 7A).^104^ This reaction can be followed by UV-vis spectroscopy at −40 °C. Isotopic labelling studies with ^18^O_2_ show an O–O stretch by Raman spectroscopy that provides good support for the Fe^3+^-hydroperoxo assignment. Additionally, IR spectroscopy of deuterium labelled N–H complex followed by reaction with O_2_ shows that the signals associated with the N–H and O–H stretches in the Fe^3+^-hydroperoxo complex originate from the ligand scaffold and hydroperoxo moiety respectively. This Fe complex can then release H_2_O_2_ upon warming. Alternatively, if this complex is instead warmed in the presence of triphenylphosphine (PPh_3_) one equivalent of triphenylphosphine oxide (OPPh_3_) can form. It was also found that the Fe^3+^-hydroperoxo complex will perform a net H-atom abstraction followed by O-atom transfer with the toluene solvent to form benzaldehyde. While only 0.131 equivalents of benzaldehyde could be detected by gas chromatography-mass spectrometry (GC-MS), this reaction demonstrates how the combination of redox-active ligands and pendent protons can enable the reductive activation of O_2_ for both C–H activation as well as O-atom transfer.

A second example of O-atom transfer *via* metal–ligand cooperativity with these DHP systems features a Ni^2+^ T-shaped complex with a ligand radical (Fig. 7B).^105^ Exposure of this complex to O_2_ results in the formation of a Ni^2+^-superoxo complex with an oxidized DHP ligand. In this case, oxidation occurs at the ligand rather than at the metal center upon O_2_ binding, paralleling the reactivity proposed in quercetin dioxygenase.^106,107^ Unlike the above-mentioned Fe^3+^-hydroperoxo complex with a DHP ligand radical, the Ni^2+^-superoxo complex utilized ligand-based electron transfer only, instead of both proton and electron transfer. This superoxo intermediate is moderately stable at room temperature. While this Ni^2+^-superoxo complex can perform stoichiometric O-atom transfer to PPh_3_, it must be heated to elicit H-atom abstraction from a C–H bond and subsequent O-atom transfer to toluene. The Ni^2+^-superoxo complex can also perform alcohol oxidation of benzyl alcohol, but once again requires heat for reactivity to occur. This is a stark contrast to the previously discussed Fe complex which could perform these reactions at low temperature or room temperature respectively. Unfortunately, the exact mechanism of oxidation for both the Fe and the Ni complexes is difficult to conclusively determine due to decomposition of the metal complex during reactivity.

The above examples with the DHP scaffold on Fe and Ni resulted in only stoichiometric reactivity. However, moving late in the first-row to Cu results in aerobic catalysis. A related Cu^2+^-superoxo complex with an oxidized and closed-shell DHP ligand can be synthesized and characterized with a suite of spectroscopic methods (Fig. 7C). This superoxo complex engages in a variety of catalytic aerobic reactivity including deformylation of 2-phenylpropionaldehyde, dehydrogenation of N–H and O–H bonds, and O-atom transfer to PPh_3_.^108^ As such, this Cu complex displays the most robust and versatile oxidative reactivity among these DHP ligated complexes. All three examples demonstrate how metal–ligand cooperativity can stabilize metal–oxygen intermediates that serve as potent oxidants. However, only the Fe(DHP) complex has provided direct evidence, *via* spectroscopy, of how ligand-based hydrogen equivalents can be transferred to O_2_. Other first row transition metals featuring the DHP ligand, including Ni and Co, have been shown to participate in hydrogenation where a more direct role for the storage of H-equivalents on the ligand is invoked.^103,109^ An interesting hypothesis is that the DHP ligand plays an active role in accepting protons or H-atoms during the C–H, N–H, or O–H activation step of various oxidations, but additional studies will be required to test this proposal. Regardless, a key conclusion from this series is how different oxidative reactivity is realized with different metal centers all on the same DHP ligand. This highlights how cooperativity between the metal and the ligand is essential beyond the fundamental design of the ligand scaffold itself.

While O-atom transfer to an outside substrate can be extremely challenging, metal–ligand cooperativity can more commonly facilitate intramolecular O-atom transfer, for instance in degradation processes. O-atom transfer to the ligand is often more accessible as the ligand as a substrate is held in close proximity to the activated O-atom equivalents. Unfortunately, it also provides a parasitic pathway that frequently precludes the possibility of performing catalytic oxidations of external substrates. These examples still provide insights into how metal–ligand cooperativity enables the activation of O_2_ for oxidative chemistry. As such, a few examples of ligand oxidation are described here. As a final note, while initial efforts in this area have focused on designing ligands for controlling the storage/flow of electrons and protons, realizing scaffolds which are oxidatively robust is an increasingly important hurdle for broader catalytic applications (as discussed below).

**Fig. 8:**
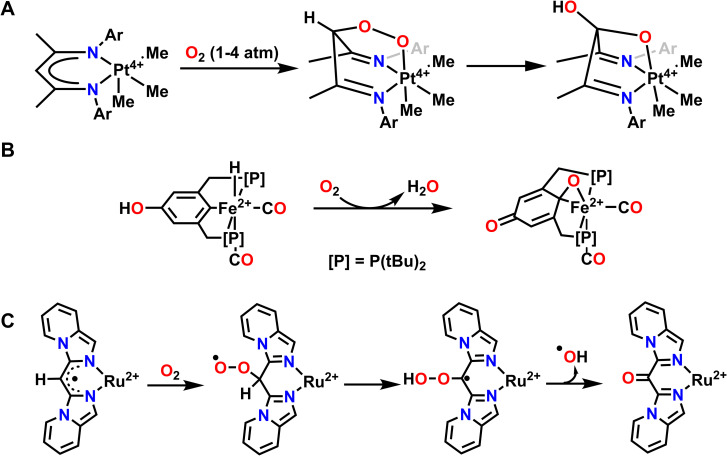
Examples where exposure to O_2_ results in O-atom transfer to the ligand *via* metal–ligand cooperativity where the ligand donates protons and electrons for the reduction of O_2_. (A), ref. 110; (B), ref. 114; (C), ref. 119.

A few examples of intramolecular O-atom transfer have been demonstrated by Karen Goldberg and coworkers. Here, the authors have explored the reactivity of Pt complexes featuring redox active ligands with O_2_ and they have observed oxidation of the ligand *via* C–O bond formation to one O-atom and coordination to the Pt center through the second O-atom to form a 6-member metallocycle ring (Fig. 8A). This Pt–peroxide complex then undergoes O–O bond cleavage to form a 5-membered metallocycle ring with one O-atom and insertion of the other O-atom into a ligand C–H bond. As such, the ligand of this complex stabilizes the coordination of O_2_ and subsequently engages in O-atom transfer to the ligand *via* insertion into a C–H bond.^110,111^ Net C–H activation of the ligand has been previously observed when reactive metal–oxygen complexes are generated.^112,113^ However, we will not discuss these other examples in detail since the reactive complex in question was not generated directly from O_2_.

Another example of O_2_ activation and subsequent reactivity with the ligand comes from Milstein and coworkers. Reaction of an Fe^2+^ hydride complex supported by a PCP pincer ligand results in hydrogenation of one oxygen equivalent of O_2_ to H_2_O and insertion of the second oxygen into the Fe–C ligand bond (Fig. 8B). A proton from the ligand backbone O–H and the hydride ligand are the hydrogen sources for this net hydrogenation reaction to form water. This results in an overall oxidized PCP ligand and one equivalent of H_2_O from the net reaction with O_2_.^114^ This reactivity is reminiscent of extradiol-cleaving catechol dioxygenases, which use the substrate as the ligand when activating O_2_. In particular, the mechanism for these dioxygenases invokes an Fe–oxygen–ligand metallocycle prior to O-atom insertion into the ligand and the subsequent reduction of the second O-atom to water.^115^ A similar O-atom insertion into a ligand C–H bond and oxidation of the auxiliary ligand, in this case trimethylphosphine (PMe_3_) to trimethylphosphine oxide (OPMe_3_), is also observed with a DHP Ni complex related to those discussed above.^116^ Abakumov and coworkers have also reported an Sb^5+^ complex that can coordinate O_2_, then undergo insertion of the terminal O-atom into the ligand to form a metallocycle and dearomatize the ligand scaffold. This reaction is reversible, and under moderate heating will reform the original Sb^5+^ complex and O_2_.^117,118^

In both the Pt and Fe examples, O_2_ is first coordinated to the metal center before proceeding through O-atom transfer to the ligand and hydrogenation. However, metal–ligand cooperativity can facilitate other O_2_ binding events, particularly through interactions with ligand-based radicals. A good example of this interaction can be observed with a Ru^2+^ complex reported by Lahiri and coworkers. Here, O_2_ initially reacts with a ligand-based radical to form a C–O–O bond, where a radical primarily resides on the terminal oxygen and effectively dearomatizes the ligand (Fig. 8C). This complex then undergoes a net 1,3-H-atom shift to rearomatize the ligand and provide a hydroperoxo intermediate. Finally, O–O bond homolysis results in the release of hydroxyl radical and overall O-atom transfer to the ligand. Importantly, this net reactivity is again enabled by metal ligand cooperativity between the Ru^2+^ center and the pincer supporting ligand.^119^

## 5 Opportunities and challenges

Metal–ligand cooperativity, particularly with a scaffold capable of storing both electron and proton equivalents, is a powerful tool in synthetic chemistry. This strategy provides a readily available source of formal H-atoms which can facilitate specific catalytic steps or the formation of various intermediates. Combining this approach to metal–ligand cooperativity with O_2_ in overall aerobic reactivity can allow for oxidative reactivity that otherwise requires significantly more harsh conditions or more oxidizing reagents to realize. While this field holds significant potential, there are also a variety of challenges that must be addressed in to effectively harness this strategy for a wide array of applications.

### 5.1 Positioning of H-atom equivalents to target desired oxygenated intermediates

The proximity of proton, H-atom, or hydride equivalents within the secondary sphere to a bound O_2_ or substrate molecule can have a dramatic, and even determinative, effect on the selectivity for various activated intermediates. As such, the positioning of these hydrogen equivalents in the secondary sphere or through cocatalysts must be harnessed to achieve the desired selectivity. Lessons from biological systems can be used for initial insights,^120–123^ but more detailed studies on differential reactivity with various placements of H-equivalents are needed. However, small changes within the secondary sphere can also result in significantly different electronic effects which may also influence the complex's ability to effectively interact with O_2_ and desired substrates. Careful balancing of ligand properties is essential for robust and effective catalysts.

### 5.2 Balancing and managing the simultaneous flow of reducing and oxidizing equivalents

The activation of O_2_ requires the addition of reducing (H-atom) equivalents, but these same reducing equivalents may also result in quenching of any active oxidizing intermediates. Thus, finding ways to balance the flow of reducing equivalents is vital to catalytic turnover. Nature circumvents this challenge by gating the delivery of proton and electron equivalents at specific points during turnover. However, this level of control is much more difficult in synthetic systems. There has been some success in this area towards alcohol oxidation, particularly primary alcohol oxidation, where the substrate can also serve as a reductant to activate O_2_. However, similar progress has been more limited with other oxidative reactions where the substrate is more difficult to oxidize. Management of both reducing and oxidizing equivalents in more challenging oxidative reactions will be vital for expanding the utility of this strategy.

### 5.3 Realizing holistic design principles for broad applicability and scope

While the design of elaborate ligand scaffolds can be useful for a specific reaction, realizing general aerobic catalysts often requires simple and modular systems. Future work will need to balance these two approaches to ensure a useful reaction scope can be achieved. An elaborate ligand design that only catalyses one reaction will rarely be widely useful in industry or academia. In this context, the above-mentioned cases with an external “cooperative” ligand such as aminoxyl radicals are noteworthy. Alternatively, future studies may find success in utilizing more robust and modular “cooperative” ligands that can donate and accept both protons and electrons to enable flexibility and broad scope that are needed for useful synthetic methodologies.

### 5.4 Building in sufficient oxidative stability to enable efficient turnover

Some of the most challenging aerobic oxidations also involve substrates which are similar to ligand scaffolds (*i.e.* aliphatic C–H groups). Thus, designing appropriate flanking groups on ligands is important to preclude intramolecular decomposition. Examples of this intramolecular decomposition were presented in the final section of this perspective. While those examples provided important evidence for how metal–ligand cooperativity can enable O-atom transfer to C–H bonds, they also demonstrated how intramolecular oxidation of the ligand scaffold precludes the possibility of catalysis. One possible avenue of investigation on this front includes replacing C–H bonds in the ligand framework with more oxidatively robust groups such as C–F bonds or oxygenated groups (*i.e.* sulfonates, carbonyls, *etc.*). However, maintaining optimized cooperative reactivity with such oxidatively robust flanking groups is a notable synthetic challenge.

## 6 Conclusions

Significant progress has been made using metal–ligand cooperativity and O_2_ for oxidative chemistry, but there is still a great deal of space for improvement in this area. In particular, there is a need to build ligand scaffolds that balance stabilizing the active oxidant and providing sufficient activation of the active oxidant to enable catalytic reactivity. Furthermore, a balance between building a ligand scaffold that minimizes intramolecular oxidation while also providing coordination and reaction space for a wide range of substrates to bind and undergo oxidation must be found. Currently, much of the research in this area functions as fundamental chemistry research, which demonstrates the viability of the approach but lacks applicability to a wide substrate scope. Future work should focus on bridging metal–ligand cooperativity in conjunction with O_2_ with the field of organic methodology to make transition metal complexes incorporating metal–ligand cooperativity an essential part of a synthetic chemist's toolkit. While this will require further fundamental studies on the principles that govern cooperative activation of O_2_, more applied investigations into tuning these systems for catalysis is also needed.

## Author contributions

Both authors contributed equally to this work.

## Conflicts of interest

There are no conflicts to declare.

## References

[cit1] Pegis M. L., Roberts J. A. S., Wasylenko D. J., Mader E. A., Appel A. M., Mayer J. M. (2015). Standard Reduction Potentials for Oxygen and Carbon Dioxide Couples in Acetonitrile and N,N-Dimethylformamide. Inorg. Chem..

[cit2] Appleby A. J. , Encyclopedia of Electrochemical Power Sources, 2009, pp. 810–852

[cit3] Agarwal R. G., Coste S. C., Groff B. D., Heuer A. M., Noh H., Parada G. A., Wise C. F., Nichols E. M., Warren J. J., Mayer J. M. (2022). Free Energies of Proton-Coupled Electron Transfer Reagents and Their Applications. Chem. Rev..

[cit4] Stille J. K., Divakaruni R. (1979). Mechanism of the Wacker Process. Stereochemistry of the Hydroxypalladation. J. Organomet. Chem..

[cit5] Keith J. A., Henry P. M. (2009). The Mechanism of the Wacker Reaction: A Tale of Two Hydroxypalladations. Angew. Chem., Int. Ed..

[cit6] Smidt J., Hafner W., Jira R., Sedlmeier J., Sieber R., Rüttinger R., Kojer H. (1959). Katalytische Umsetzungen von Olefinen an Platinmetall-Verbindungen Das Consortium-Verfahren zur Herstellung von Acetaldehyd. Angew. Chem..

[cit7] Anson C. W., Ghosh S., Hammes-Schiffer S., Stahl S. S. (2016). Co(salophen)-Catalyzed Aerobic Oxidation of p-Hydroquinone: Mechanism and Implications for Aerobic Oxidation Catalysis. J. Am. Chem. Soc..

[cit8] Bates J. S., Biswas S., Suh S. E., Johnson M. R., Mondal B., Root T. W., Stahl S. S. (2022). Chemical and Electrochemical O_2_ Reduction on Earth-Abundant M-N-C Catalysts and Implications for Mediated Electrolysis. J. Am. Chem. Soc..

[cit9] Anson C. W., Stahl S. S. (2017). Cooperative Electrocatalytic O_2_ Reduction Involving Co(salophen) with p-Hydroquinone as an Electron-Proton Transfer Mediator. J. Am. Chem. Soc..

[cit10] Gerken J. B., Stahl S. S. (2015). High-Potential Electrocatalytic O_2_ Reduction with Nitroxyl/NOx Mediators: Implications for Fuel Cells and Aerobic Oxidation Catalysis. ACS Cent. Sci..

[cit11] Tereniak S. J., Landis C. R., Stahl S. S. (2018). Are Phosphines Viable Ligands for Pd-Catalyzed Aerobic Oxidation Reactions? Contrasting Insights from a Survey of Six Reactions. ACS Catal..

[cit12] Rafiee M., Konz Z. M., Graaf M. D., Koolman H. F., Stahl S. S. (2018). Electrochemical Oxidation of Alcohols and Aldehydes to Carboxylic Acids Catalyzed by 4-Acetamido-TEMPO: An Alternative to ‘anelli’ and ‘pinnick’ Oxidations. ACS Catal..

[cit13] Singh K. K. and Garcia-Bosch I., in Redox - Active Ligands, Wiley, 2024, pp. 175–195

[cit14] Wertz S., Studer A. (2013). Nitroxide-Catalyzed Transition-Metal-Free Aerobic Oxidation Processes. Green Chem..

[cit15] Wang D., Weinstein A. B., White P. B., Stahl S. S. (2018). Ligand-Promoted Palladium-Catalyzed Aerobic Oxidation Reactions. Chem. Rev..

[cit16] Ma Z., Mahmudov K. T., Aliyeva V. A., Gurbanov A. V., Pombeiro A. J. L. (2020). TEMPO in Metal Complex Catalysis. Coord. Chem. Rev..

[cit17] Leifert D., Studer A. (2023). Organic Synthesis Using Nitroxides. Chem. Rev..

[cit18] Schmidt V. A., Alexanian E. J. (2010). Metal-Free, Aerobic Dioxygenation of Alkenes Using Hydroxamic Acids. Angew. Chem., Int. Ed..

[cit19] Wang X., Wang D. Z. (2011). Aerobic Oxidation of Secondary Benzylic Alcohols and Direct Oxidative Amidation of Aryl Aldehydes Promoted by Sodium Hydride. Tetrahedron.

[cit20] Sheldon R. A., Arenas I. W. C. E. (2004). Organocatalytic Oxidations Mediated by Nitroxyl Radicals. Adv. Synth. Catal..

[cit21] Schmidt V. A., Alexanian E. J. (2012). Metal-Free, Aerobic Ketooxygenation of Alkenes Using Hydroxamic Acids. Chem. Sci..

[cit22] Giglio B. C., Schmidt V. A., Alexanian E. J. (2011). Metal-Free, Aerobic Dioxygenation of Alkenes Using Simple Hydroxamic Acid Derivatives. J. Am. Chem. Soc..

[cit23] Lv G., Wang H., Yang Y., Deng T., Chen C., Zhu Y., Hou X. (2015). Graphene Oxide: A Convenient Metal-Free Carbocatalyst for Facilitating Aerobic Oxidation of 5-Hydroxymethylfurfural into 2, 5-Diformylfuran. ACS Catal..

[cit24] Chen K., Xie H. (2017). Selective Aerobic Oxidation Promoted by Highly Efficient Multi-Nitroxy Organocatalysts. Chin. J. Catal..

[cit25] Li L., Zhao Y. L., Wang Q., Lin T., Liu Q. (2015). Base-Promoted Oxidative C-H Functionalization of α-Amino Carbonyl Compounds Under Mild Metal-Free Conditions: Using Molecular Oxygen as the Oxidant. Org. Lett..

[cit26] Romero E., Gómez Castellanos J. R., Gadda G., Fraaije M. W., Mattevi A. (2018). Same Substrate, Many Reactions: Oxygen Activation in Flavoenzymes. Chem. Rev..

[cit27] Rolff M., Schottenheim J., Decker H., Tuczek F. (2011). Copper-O_2_ Reactivity of Tyrosinase Models Towards External Monophenolic Substrates: Molecular Mechanism and Comparison with the Enzyme. Chem. Soc. Rev..

[cit28] Fiedler A. T., Fischer A. A. (2017). Oxygen Activation by Mononuclear Mn, Co, and Ni Centers in Biology and Synthetic Complexes. J. Biol. Inorg. Chem..

[cit29] Solomon E. I., Light K. M., Liu L. V., Srnec M., Wong S. D. (2013). Geometric and Electronic Structure Contributions to Function in Non-Heme Iron Enzymes. Acc. Chem. Res..

[cit30] Lange S. J., Clue L. (1998). Oxygen Activating Nonheme Iron Enzymes. Curr. Opin. Chem. Biol..

[cit31] Newcomb M., Hollenberg P. F., Coon M. J. (2003). Multiple Mechanisms and Multiple Oxidants in P450-Catalyzed Hydroxylations. Arch. Biochem. Biophys..

[cit32] Hamdane D., Zhang H., Hollenberg P. (2008). Oxygen Activation by Cytochrome P450 Monooxygenase. Photosynth. Res..

[cit33] Jasniewski A. J., Que L. (2018). Dioxygen Activation by Nonheme Diiron Enzymes: Diverse Dioxygen Adducts, High-Valent Intermediates, and Related Model Complexes. Chem. Rev..

[cit34] Manna S., Kong W. J., Bäckvall J. E. (2021). Adv. Catal..

[cit35] Gordon Z., Miller T. J., Leahy C. A., Matson E. M., Burgess M., Drummond M. J., Popescu C. V., Smith C. M., Lord R. L., Rodríguez-López J., Fout A. R. (2019). Characterization of Terminal Iron(III)-Oxo and Iron(III)-Hydroxo Complexes Derived from O_2_ Activation. Inorg. Chem..

[cit36] Hill E. A., Weitz A. C., Onderko E., Romero-Rivera A., Guo Y., Swart M., Bominaar E. L., Green M. T., Hendrich M. P., Lacy D. C., Borovik A. S. (2016). Reactivity of an Fe^IV^-Oxo Complex with Protons and Oxidants. J. Am. Chem. Soc..

[cit37] Cook S. A., Borovik A. S. (2015). Molecular Designs for Controlling the Local Environments around Metal Ions. Acc. Chem. Res..

[cit38] Moore C. M., Szymczak N. K. (2015). Redox-Induced Fluoride Ligand Dissociation Stabilized by Intramolecular Hydrogen Bonding. Chem. Commun..

[cit39] Pegis M. L., Wise C. F., Martin D. J., Mayer J. M. (2018). Oxygen Reduction by Homogeneous Molecular Catalysts and Electrocatalysts. Chem. Rev..

[cit40] Liao Q., Liu T., Johnson S. I., Klug C. M., Wiedner E. S., Morris Bullock R., Dubois D. L. (2019). Evaluation of Attractive Interactions in the Second Coordination Sphere of Iron Complexes Containing Pendant Amines. Dalton Trans..

[cit41] Loewen N. D., Thompson E. J., Kagan M., Banales C. L., Myers T. W., Fettinger J. C., Berben L. A. (2016). A Pendant Proton Shuttle on [Fe_4_N(CO)_12_]^−^ Alters Product Selectivity in Formate vs. H_2_ Production via the Hydride [H-Fe4N(CO)_12_]^−^. Chem. Sci..

[cit42] MacLeod K. C., Lewis R. A., DeRosha D. E., Mercado B. Q., Holland P. L. (2017). C−H and C−N Activation at Redox-Active Pyridine Complexes of Iron. Angew. Chem., Int. Ed..

[cit43] Zell T., Milstein D. (2015). Hydrogenation and Dehydrogenation Iron Pincer Catalysts Capable of Metal-Ligand Cooperation by Aromatization/Dearomatization. Acc. Chem. Res..

[cit44] Rauch M., Kar S., Kumar A., Avram L., Shimon L. J. W., Milstein D. (2020). Metal-Ligand Cooperation Facilitates Bond Activation and Catalytic Hydrogenation with Zinc Pincer Complexes. J. Am. Chem. Soc..

[cit45] Dubois M. R., Dubois D. L. (2009). Development of Molecular Electrocatalysts for CO_2_ Reduction and H_2_ Production/Oxidation. Acc. Chem. Res..

[cit46] Pegis M. L., Martin D. J., Wise C. F., Brezny A. C., Johnson S. I., Johnson L. E., Kumar N., Raugei S., Mayer J. M. (2020). Mechanism of Catalytic O_2_ Reduction by Iron Tetraphenylporphyrin. J. Am. Chem. Soc..

[cit47] Broere D. L. J., Van Leest N. P., De Bruin B., Siegler M. A., Van Der Vlugt J. I. (2016). Reversible Redox Chemistry and Catalytic C(sp^3^)-H Amination Reactivity of a Paramagnetic Pd Complex Bearing a Redox-Active o-Aminophenol-Derived NNO Pincer Ligand. Inorg. Chem..

[cit48] Harris C. F., Bayless M. B., Van Leest N. P., Bruch Q. J., Livesay B. N., Bacsa J., Hardcastle K. I., Shores M. P., De Bruin B., Soper J. D. (2017). Redox-Active Bis(phenolate) N-Heterocyclic Carbene [OCO] Pincer Ligands Support Cobalt Electron Transfer Series Spanning Four Oxidation States. Inorg. Chem..

[cit49] Chatterjee S., Paine T. K. (2023). Dioxygen Reduction and Bioinspired Oxidations by Non-heme Iron(II)−α-Hydroxy Acid Complexes. Acc. Chem. Res..

[cit50] Luca O. R., Crabtree R. H. (2013). Redox-active ligands in catalysis. Chem. Soc. Rev..

[cit51] Wong J. L., Sánchez R. H., Logan J. G., Zarkesh R. A., Ziller J. W., Heyduk A. F. (2013). Disulfide Reductive Elimination from an Iron(iii) Complex. Chem. Sci..

[cit52] Arevalo R., Chirik P. J. (2019). Enabling Two-Electron Pathways with Iron and Cobalt: From Ligand Design to Catalytic Applications. J. Am. Chem. Soc..

[cit53] Bagh B., Broere D. L. J., Sinha V., Kuijpers P. F., Van Leest N. P., De Bruin B., Demeshko S., Siegler M. A., Van Der Vlugt J. I. (2017). Catalytic Synthesis of N-Heterocycles via Direct C(sp^3^ )-H Amination Using an Air-Stable Iron(III) Species with a Redox-Active Ligand. J. Am. Chem. Soc..

[cit54] Sinha S., Das S., Sikari R., Parua S., Brandaõ P., Demeshko S., Meyer F., Paul N. D. (2017). Redox Noninnocent Azo-Aromatic Pincers and Their Iron Complexes. Isolation, Characterization, and Catalytic Alcohol Oxidation. Inorg. Chem..

[cit55] Ott J. C., Bürgy D., Guan H., Gade L. H. (2022). 3d Metal Complexes in T-shaped Geometry as a Gateway to Metalloradical Reactivity. Acc. Chem. Res..

[cit56] Lu X., Wang S., Qin J. H. (2022). Isolating Fe-O_2_ Intermediates in Dioxygen Activation by Iron Porphyrin Complexes. Molecules.

[cit57] Liu T., Liao Q., O'Hagan M., Hulley E. B., DuBois D. L., Bullock R. M. (2015). Iron Complexes Bearing Diphosphine Ligands with Positioned Pendant Amines as Electrocatalysts for the Oxidation of H_2_. Organometallics.

[cit58] Lyaskovskyy V., De Bruin B. (2012). Redox Non-innocent Ligands: Versatile new Tools to Control Catalytic Reactions. ACS Catal..

[cit59] Kaim W. (2011). Manifestations of Noninnocent Ligand Behavior. Inorg. Chem..

[cit60] Jesse K. A., Chang M. C., Filatov A. S., Anderson J. S. (2021). Iron(II) Complexes Featuring a Redox-Active Dihydrazonopyrrole Ligand. Z. Anorg. Allg. Chem..

[cit61] Horak K. T., Agapie T. (2016). Dioxygen Reduction by a Pd(0)-Hydroquinone Diphosphine Complex. J. Am. Chem. Soc..

[cit62] Henthorn J. T., Agapie T. (2015). Combination of Redox-Active Ligand and Lewis Acid for Dioxygen Reduction with π-Bound Molybdenum-Quinonoid Complexes. J. Am. Chem. Soc..

[cit63] Senft L., Moore J. L., Franke A., Fisher K. R., Scheitler A., Zahl A., Puchta R., Fehn D., Ison S., Sader S., Ivanović-Burmazović I., Goldsmith C. R. (2021). Quinol-Containing Ligands Enable High Superoxide Dismutase Activity by Modulating Coordination Number, Charge, Oxidation States and Stability of Manganese Complexes Throughout Redox Cycling. Chem. Sci..

[cit64] Ward M. B., Scheitler A., Yu M., Senft L., Zillmann A. S., Gorden J. D., Schwartz D. D., Ivanović-Burmazović I., Goldsmith C. R. (2018). Superoxide Dismutase Activity Enabled by a Redox-Active Ligand Rather than Metal. Nat. Chem..

[cit65] Obisesan S. V., Rose C., Farnum B. H., Goldsmith C. R. (2022). Co(II) Complex with a Covalently Attached Pendent Quinol Selectively Reduces O_2_ to H_2_O. J. Am. Chem. Soc..

[cit66] Hooe S. L., Cook E. N., Reid A. G., Machan C. W. (2021). Non-Covalent Assembly of Proton Conors and p-Benzoquinone Anions for Co-Electrocatalytic Reduction of Dioxygen. Chem. Sci..

[cit67] Borovik A. S. (2005). Bioinspired Hydrogen Bond Motifs in Ligand Design: The Role of Noncovalent Interactions in Metal Ion Mediated Activation of Dioxygen. Acc. Chem. Res..

[cit68] Drummond M. J., Ford C. L., Gray D. L., Popescu C. V., Fout A. R. (2019). Radical Rebound Hydroxylation Versus H-Atom Transfer in Non-Heme Iron(III)-Hydroxo Complexes: Reactivity and Structural Differentiation. J. Am. Chem. Soc..

[cit69] Dahl E. W., Kiernicki J. J., Zeller M., Szymczak N. K. (2018). Hydrogen Bonds Dictate O_2_ Capture and Release within a Zinc Tripod. J. Am. Chem. Soc..

[cit70] Shook R. L., Peterson S. M., Greaves J., Moore C., Rheingold A. L., Borovik A. S. (2011). Catalytic Reduction of Dioxygen to Water with a Monomeric Manganese Complex at Room Temperature. J. Am. Chem. Soc..

[cit71] Sagar K., Kim M., Wu T., Zhang S., Bominaar E. L., Siegler M. A., Hendrich M., Garcia-Bosch I. (2024). Mimicking the Reactivity of LPMOs with a Mononuclear Cu Complex. Eur. J. Inorg. Chem..

[cit72] Jiang Y., Telser J., Goldberg D. P. (2009). Evidence for the Formation of a Mononuclear Ferric-Hydroperoxo Complex via the Reaction of Dioxygen with an (N_4_S(thiolate))iron(II) Complex. Chem. Commun..

[cit73] Pinto A. F., Rodrigues J. V., Teixeira M. (2010). Reductive Elimination of Superoxide: Structure and Mechanism of Superoxide Reductases. Biochim. Biophys. Acta, Proteins Proteomics.

[cit74] Meunier B., de Visser S. P., Shaik S. (2004). Mechanism of Oxidation Reactions Catalyzed by Cytochrome P450 Enzymes. Chem. Rev..

[cit75] Kal S., Que L. (2019). Activation of a Non-Heme Fe III -OOH by a Second Fe III to Hydroxylate Strong C−H Bonds: Possible Implications for Soluble Methane Monooxygenase. Angew. Chem..

[cit76] Nam E., Alokolaro P. E., Swartz R. D., Gleaves M. C., Pikul J., Kovacs J. A. (2011). Investigation of the Mechanism of Gormation of a Thiolate-Ligated Fe(III)-OOH. Inorg. Chem..

[cit77] Kitagawa T., Dey A., Lugo-Mas P., Benedict J. B., Kaminsky W., Solomon E., Kovacs J. A. (2006). A Functional Model for the Cysteinate-Ligated Non-Heme Iron Enzyme Superoxide Reductase (SOR). J. Am. Chem. Soc..

[cit78] Xu S., Veach J. J., Oloo W. N., Peters K. C., Wang J., Perry R. H., Que L. (2018). Detection of a Transient Fe^V^(O)(OH) Species Involved in Olefin Oxidation by a Bio-Inspired Non-Haem Iron Catalyst. Chem. Commun..

[cit79] Serrano-Plana J., Acuña-Parés F., Dantignana V., Oloo W. N., Castillo E., Draksharapu A., Whiteoak C. J., Martin-Diaconescu V., Basallote M. G., Luis J. M., Que L., Costas M., Company A. (2018). Acid-Triggered O−O Bond Heterolysis of a Nonheme Fe^III^(OOH) Species for the Stereospecific Hydroxylation of Strong C−H Bonds. Chem.–Eur. J..

[cit80] Kovacs J. A., Brines L. M. (2007). Understanding how the Thiolate Sulfur Contributes to the Function of the Non-Heme Iron Enzyme Superoxide Reductase. Acc. Chem. Res..

[cit81] Simaan A. J., Banse F., Girerd J. J., Wieghardt K., Bill E. (2001). The Electronic Structure of Non-Heme Iron(III)-Hydroperoxo and Iron(III)-Peroxo Model Complexes Studied by Mössbauer and Electron Paramagnetic Resonance Spectroscopies. Inorg. Chem..

[cit82] Li F., Meier K. K., Cranswick M. A., Chakrabarti M., Van Heuvelen K. M., Münck E., Que L. (2011). Characterization of a High-Spin Non-Heme Fe^III^-OOH Intermediate and its Quantitative Conversion to an Fe^IV^=O Complex. J. Am. Chem. Soc..

[cit83] Wegeberg C., Browne W. R., McKenzie C. J. (2019). Cis Donor Influence on O-O Bond Lability in Iron(III) Hydroperoxo Complexes: Oxidation Catalysis and Ligand Transformation. Inorg. Chem..

[cit84] Hong S., Lee Y. M., Shin W., Fukuzumi S., Nam W. (2009). Dioxygen Activation by Mononuclear Nonheme Iron(II) Complexes Generates Iron-Oxygen Intermediates in the Presence of an NADH Analogue and Proton. J. Am. Chem. Soc..

[cit85] Panda S., Phan H., Dunietz E. M., Brueggemeyer M. T., Hota P. K., Siegler M. A., Jose A., Bhadra M., Solomon E. I., Karlin K. D. (2024). Intramolecular Phenolic H-Atom Abstraction by a N_3_ ArOH Ligand-Supported (μ-η^2^:η^2^ -Peroxo)dicopper(II) Species Relevant to the Active Site Function of oxy-Tyrosinase. J. Am. Chem. Soc..

[cit86] Kipouros I., Stanczak A., Ginsbach J. W., Andrikopoulos P. C., Rulısek L., Solomon E. I. (2022). Elucidation of the Tyrosinase/O_2_/Monophenol Ternary Intermediate that Dictates the Monooxygenation Mechanism in Melanin Biosynthesis. Proc. Natl. Acad. Sci. U. S. A..

[cit87] Kipouros I., Stańczak A., Dunietz E. M., Ginsbach J. W., Srnec M., Rulíšek L., Solomon E. I. (2023). Experimental Evidence and Mechanistic Description of the Phenolic H-Transfer to the Cu_2_O_2_ Active Site of oxy-Tyrosinase. J. Am. Chem. Soc..

[cit88] Rajabimoghadam K., Darwish Y., Bashir U., Pitman D., Eichelberger S., Siegler M. A., Swart M., Garcia-Bosch I. (2018). Catalytic Aerobic Oxidation of Alcohols by Copper Complexes Bearing Redox-Active Ligands with Tunable H-Bonding Groups. J. Am. Chem. Soc..

[cit89] Charette B. J., Ziller J. W., Heyduk A. F. (2021). Exploring Ligand-Centered Hydride and H-Atom Transfer. Inorg. Chem..

[cit90] Hollas A. M., Ziller J. W., Heyduk A. F. (2018). Three Oxidation States of the Bis(3,5-di-tert-butyl-2-phenolato)azanido Pincer Ligand on Chromium(III). Polyhedron.

[cit91] Charette B. J., Ziller J. W., Heyduk A. F. (2021). Metal-Ion Influence on Ligand-Centered Hydrogen-Atom Transfer. Inorg. Chem..

[cit92] Johnson R. A. and Sharpless K. B., Comprehensive Organic Synthesis, 1991, vol. 7, pp. 389–436

[cit93] Wang Y., Dubois J. L., Hedman B., Hodgson K. O., Stack T. D. P. (1998). Catalytic Galactose Oxidase Models: Biomimetic Cu(II)-Phenoxyl-Radical Reactivity. Science.

[cit94] Chaudhuri P., Hess M., Müller J., Hildenbrand K., Bill E., Weyhermüller T., Wieghardt K. (1999). Aerobic Oxidation of Primary Alcohols (Including Methanol) by Copper(II)- and Zinc(N)-Phenoxyl Radical Catalysts. J. Am. Chem. Soc..

[cit95] Chaudhuri P., Hess M., Weyhermüller T., Wieghardt K. (1999). Aerobic Oxidation of Primary Alcohols by a New Mononuclear CuII-Radical Catalyst. Angew. Chem., Int. Ed..

[cit96] Markó I. E., Giles P. R., Tsukazaki M., Brown S. M., Urch C. J. (1996). Copper-Catalyzed Oxidation of Alcohols to Aldehydes and Ketones: An Efficient, Aerobic Alternative. Science.

[cit97] Markó I. E., Gautier A., Chellé-Regnaut I., Giles P. R., Tsukazaki M., Urch C. J., Brown S. M. (1998). Efficient and Practical Catalytic Oxidation of Alcohols Using Molecular Oxygen. J. Org. Chem..

[cit98] Hoover J. M., Ryland B. L., Stahl S. S. (2013). Mechanism of Copper(I)/TEMPO-Catalyzed Aerobic Alcohol Oxidation. J. Am. Chem. Soc..

[cit99] Walroth R. C., Miles K. C., Lukens J. T., MacMillan S. N., Stahl S. S., Lancaster K. M. (2017). Electronic Structural Analysis of Copper(II)-TEMPO/ABNO Complexes Provides Evidence for Copper(I)-Oxoammonium Character. J. Am. Chem. Soc..

[cit100] Nutting J. E., Mao K., Stahl S. S. (2021). Iron(III) Nitrate/TEMPO-Catalyzed Aerobic Alcohol Oxidation: Distinguishing between Serial versus Integrated Redox Cooperativity. J. Am. Chem. Soc..

[cit101] Sinha S., Das S., Mondal R., Mandal S., Paul N. D. (2020). Cobalt Complexes of Redox Noninnocent Azo-Aromatic Pincers. Isolation, Characterization, and Application as Catalysts for the Synthesis of Quinazolin-4(3: H)-ones. Dalton Trans..

[cit102] Czaikowski M. E., Anferov S. W., Tascher A. P., Anderson J. S. (2024). Electrocatalytic Semihydrogenation of Terminal
Alkynes Using Ligand-Based Transfer of Protons and Electrons. J. Am. Chem. Soc..

[cit103] McNeece A. J., Jesse K. A., Filatov A. S., Schneider J. E., Anderson J. S. (2021). Catalytic Hydrogenation Enabled by Ligand-Based Storage of Hydrogen. Chem. Commun..

[cit104] Jesse K. A., Anferov S. W., Collins K. A., Valdez-Moreira J. A., Czaikowski M. E., Filatov A. S., Anderson J. S. (2021). Direct Aerobic Generation of a Ferric Hydroperoxo Intermediate Via a Preorganized Secondary Coordination Sphere. J. Am. Chem. Soc..

[cit105] Mcneece A. J., Jesse K. A., Xie J., Filatov A. S., Anderson J. S. (2020). Generation and Oxidative Reactivity of a Ni(II) Superoxo Complex via Ligand-Based Redox Non-Innocence. J. Am. Chem. Soc..

[cit106] Jeoung J., Nianios D., Fetzner S., Dobbek H. (2016). Quercetin-2,4-Dioxygenase Aktiviert Sauerstoff in Einem “side-on” Gebundenen O_2_ -Ni-Komplex. Angew. Chem..

[cit107] Li H., Wang X., Tian G., Liu Y. (2018). Insights into the Dioxygen Activation and Catalytic Mechanism of the Nickel-Containing Quercetinase. Catal. Sci. Technol..

[cit108] Czaikowski M. E., McNeece A. J., Boyn J.-N., Jesse K. A., Anferov S. W., Filatov A. S., Mazziotti D. A., Anderson J. S. (2022). Generation and Aerobic Oxidative Catalysis of a Cu(II) Superoxo Complex Supported by a Redox-Active Ligand. J. Am. Chem. Soc..

[cit109] Anferov S. W., Filatov A. S., Anderson J. S. (2022). Cobalt-Catalyzed Hydrogenation Reactions Enabled by Ligand-Based Storage of Dihydrogen. ACS Catal..

[cit110] Scheuermann M. L., Fekl U., Kaminsky W., Goldberg K. I. (2010). Metal-Ligand Cooperativity in O_O_ Activation: Observation of a ‘Pt-O-O-C’ Peroxo Intermediate. Organometallics.

[cit111] Scheuermann M. L., Luedtke A. T., Hanson S. K., Fekl U., Kaminsky W., Goldberg K. I. (2013). Reactions of Five-Coordinate Platinum(IV) Complexes with Molecular Oxygen. Organometallics.

[cit112] Goetz M. K., Schneider J. E., Filatov A. S., Jesse K. A., Anderson J. S. (2021). Enzyme-Like Hydroxylation of Aliphatic C-H Bonds from an Isolable Co-Oxo Complex. J. Am. Chem. Soc..

[cit113] Shi S., Wang Y., Xu A., Wang H., Zhu D., Roy S. B., Jackson T. A., Busch D. H., Yin G. (2011). Distinct Reactivity Differences of Metal Oxo and its Corresponding Hydroxo Moieties in Oxidations: Implications from a Manganese(IV) Complex having Dihydroxide Ligand. Angew. Chem., Int. Ed..

[cit114] Dauth A., Gellrich U., Diskin-Posner Y., Ben-David Y., Milstein D. (2017). The Ferraquinone-Ferrahydroquinone Couple: Combining Quinonic and Metal-Based Reactivity. J. Am. Chem. Soc..

[cit115] Costas M., Mehn M. P., Jensen M. P., Que L. (2004). Dioxygen Activation at Mononuclear Nonheme Iron Active Sites: Enzymes, Models, and Intermediates. Chem. Rev..

[cit116] Chang M. C., McNeece A. J., Hill E. A., Filatov A. S., Anderson J. S. (2018). Ligand-Based Storage of Protons and Electrons in Dihydrazonopyrrole Complexes of Nickel. Chem.–Eur. J..

[cit117] Fukin G. K., Baranov E. V., Poddel'Sky A. I., Cherkasov V. K., Abakumov G. A. (2012). Reversible Binding of Molecular Oxygen to Catecholate and Amidophenolate Complexes of SbV: Electronic and Steric Factors. ChemPhysChem.

[cit118] Abakumov G. A., Poddel’sky A. I., Grunova E. V., Cherkasov V. K., Fukin G. K., Kurskii Y. A., Abakumova L. G. (2005). Reversible Binding of Dioxygen by a Non-Transition-Metal Complex. Angew. Chem., Int. Ed..

[cit119] Panda S., Bera S. K., Goel P., Dutta A. K., Lahiri G. K. (2019). Ruthenium-Chelated Non-Innocent Bis(heterocyclo)methanides: A Mimicked β-Diketiminate. Inorg. Chem..

[cit120] Van Stappen C., Deng Y., Liu Y., Heidari H., Wang J. X., Zhou Y., Ledray A. P., Lu Y. (2022). Designing Artificial Metalloenzymes by Tuning of the Environment beyond the Primary Coordination Sphere. Chem. Rev..

[cit121] Ehudin M. A., Gee L. B., Sabuncu S., Braun A., Moënne-Loccoz P., Hedman B., Hodgson K. O., Solomon E. I., Karlin K. D. (2019). Tuning the Geometric and Electronic Structure of Synthetic High-Valent Heme Iron(IV)-Oxo Models in the Presence of a Lewis Acid and Various Axial Ligands. J. Am. Chem. Soc..

[cit122] Zhou Y., Mirts E. N., Yook S., Waugh M., Martini R., Jin Y. S., Lu Y. (2023). Reshaping the 2-Pyrone Synthase Active Site for Chemoselective Biosynthesis of Polyketides. Angew. Chem., Int. Ed..

[cit123] Ehudin M. A., Schaefer A. W., Adam S. M., Quist D. A., Diaz D. E., Tang J. A., Solomon E. I., Karlin K. D. (2019). Influence of Intramolecular Secondary Sphere Hydrogen-Bonding Interactions on Cytochrome: C Oxidase Inspired Low-Spin Heme-Peroxo-Copper Complexes. Chem. Sci..

